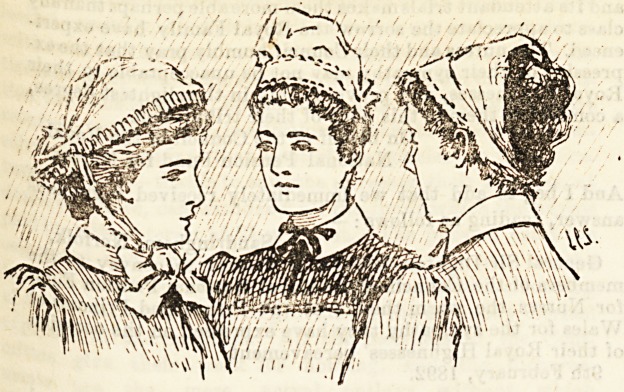# The Hospital Nursing Supplement

**Published:** 1892-03-12

**Authors:** 


"The Hospital" Nursing Mirror.

Being the Extra Nursing Supplement of "The Hospital" Newspaper.

Contributions for this Supplement should be addressed to the Editor, The Hospital, 140, Strand, London, W.C., and should have the word "Nursing" plainly written in left hand top corner of the envelope.

### En Passant.

A NURSE IN BERMUDA.—A correspondent writes that she has lately been with a patient to Bermuda. They found the climate very warm and trying, but the patient made a complete recovery. The only hospitals in Bermuda are the naval and military ones; an American visitor seeing the need of a civil hospital and trained nurses gave a sum of money to build a cottage hospital; it has been finished and is a very nice building, but has never been opened owing to the dilatoriness of the people. The population is 5,384 white people, and 8,564 coloured. They are entirely dependent on the rains for water, as there are no streams on the island which are of coral formation. The houses, roofs and all are built of blocks of coral, and look very pretty among the cedar trees.

AN INFLUENZA STORY.—A district nurse, twenty miles from London, had the following graphic description given her of how influenza was treated ten miles nearer London: "The treatment is quite different there to what it is here, miss; my son's master sent him to bed, and in the evening he came to him, and put such a curious thing in his mouth like a bit of glass tube, Jim says, only stopped up at one end. He told him to keep it there a few minutes, and it seemed, Jim says, to get quite full of black stuff, black corruption like, and it seemed to draw it all out of his mouth, and he felt himself getting better. Then the master looked at it, and all he said was, 'You keep quiet in bed awhile.' Jim had a wonderful good night; and the next morning the doctor did the same, but there wasn't hardly any of that black stuff then, it had all gone out of him, you know, and the master did not do it any more, only he kept him in bed most a fortnight; but, Jim says, it's wonderful what came out of his mouth in that tube, and how it made him feel better, getting rid of all that bad stuff. It is curious, ain't it, miss?"

POOR-LAW NURSES.—Miss Twining has written with regard to these proposed nurses, and also with regard to the non-necessity of their presence in Worthing: "I am indeed rejoiced to find the Local Government Board has at last sanctioned the plan, as we have so long desired; but may I be allowed to explain to the Guardians and ratepayers what such a scheme would involve here in this Union? If the whole extent of over fourteen miles is to be visited by nurses, there will have to be at least four or five centres, each nurse costing £60 a year, for how are district villages to be nursed upon any other plan? Then, if nurses are to retain with cases either by day or by night, how can one attend to ten or twenty in a day as at present? During the last few months over 850 visits have been paid to Poor Law cases by the Worthing district nurses. For £20 a year we could have undertaken to do all the work required, and if it became too much, a third nurse would have been engaged." The Sunderland Guardians held a special meeting to consider the order of the Local Government Board. Mr. Backhouse said his district committee when they had a case before them which they thought required nursing, always recommended the workhouse, and refused to give out-door relief. That might be said to be a test, but he considered it was the best test. For the present he thought they could not adopt the order. The order was not adopted, the chief reason being that the same day the Mayoress of Sunderland presided over a meeting for providing district nurses for the town by voluntary subscriptions.

ST. MARY'S NURSES' HOME.—This Home and Cottage Hospital, Plaistow, E., were visited on Thursday, February 25th, by the Duchess of Westminster, who is patron of the Nurses' Home. Her Grace expressed herself much pleased with all the arrangements. The patients were much gratified at seeing the Duchess, and the lovely flowers she left behind were thoroughly appreciated.

SHORT ITEMS.—The children at the Alexandra Hospital for Hip Disease had their annual treat on February 26th, when all the nursing staff helped to make the entertainment pass off satisfactorily.—Mary Ann Grainger, of Cardiff, has been fined £5 for going to a monthly case from a house in which there was scarlet fever.—Nurse Grey, of Lynn, attended 157 cases last year.—The nurse on whose behalf Miss Middleton has been appealing has been moved to her new home, and bore the journey well. She is so pleased with the change, though she speaks appreciatively of the consideration shown her at the Union Infirmary, where she has been so long.—Miss Nuttall is delivering two successful courses of nursing lectures at Wigan.—A discussion on district nursing, ending in no obvious result, has been held at the Southwark Women's Settlement. The most interesting speech was that by Miss Hughes (of Bloomsbury) in favour of ladies as district nurses.—On Tuesday night the Midwives' Bill was under discussion at the Somerville Club. Mrs. Henry Smith spoke well and Miss Rosalind Paget took the chair; the meeting was very satisfactory.—Miss Prosser, district nurse at Winchester, attended a large number of cases last year and has been thanked by the Committee for her efficient services.—The district nurses of Portsmouth attended 181 cases last year; the annual meeting was poorly attended, and Portsmouth wants waking up on this subject.—There is war between the District Nursing Association of Dukinfield and the doctors, the *causus belli* being that a lady on the committee is said to have interfered with the medical treatment of a case.

THE WORCESTER INSTITUTION.—There was a very good gathering at the late annual meeting of this institution, amongst those present being Sir Harry Vernon and the Mayor of Worcester, Mr. G. E. Matin. The Chairman moved the adoption of the report. He said that the past year had been one of exceptional sickness. It commenced with a severe outbreak of influenza, and finished with a similar outbreak. Not only was an extraordinary amount of work thrown upon the nurses, but several of the nurses themselves fell victims to the disease, and, therefore, the work they had had to do told upon them severely. The acknowledgment made in the report of the devotion shown by the Lady Superintendent and her staff was amply deserved. It was a matter for congratulation that the adverse balance of £135 had been reduced by little more than a half, and since the report was printed £36 had been received in nursing fees and £13 in arrears of subscriptions. The Chairman went on to explain that the Rural Nursing Association, with which this institution was associated, was an outcome of the Women's Jubilee Offering to the Queen. The total number of applications for nurses during the year was 460, of which 203 had to be refused, and that showed that the demand exceeded the supply to an enormous extent. The average weekly number of probationers had fallen from 7¼ to 4 5-12, and he regretted that so excellent an institution should be unable to increase its staff to meet increasing demands. He hoped the public would give such support as would not only enable the society to carry on the work it was doing, but to increase its staff and to extend its work. The Home Hospital had been successfully launched, and had already done much good work. He congratulated the institution upon the fact that their districts had increased from 13 to 19. The cases visited amounted to 425. Archdeacon Walters seconded the report, which was adopted unanimously. We congratulate the committee on the report.

### On the Nursing of Children.

### V.—SPLINTS.

We propose to consider splints entirely from the nursing side, without any attempt to trespass on the surgeon's province.

Most nurses can recall their first experience in padding a splint, and how quickly their first impression of the simplicity of the undertaking vanished as the difficulties manifested themselves one by one. Happy for them if a friendly warning prevented their manufacturing a bag, and stuffing it like a lumpy pillow, than which no more objectionable pad can possibly be imagined.

The light, soft stuffing which is now supplied, ready for use, is a great improvement on the tow which, with or without a layer of cotton wool, had once upon a time to serve for this purpose. But of whatever the "padding" may consist, it must be absolutely free from any knots, or knobs, or prickly points, it must be shaken and coaxed and humoured, until it is as light and smooth and evenly laid as is possible. Time devoted to accomplishing this is not wasted, for we must remember that this same pad will be used in one position, and will press against the delicate skin of a child for weeks, or even months.

Of course, there is a great variety of work put into the padding of splints. Those made by one nurse may look "quite lovely" to the eyes of a novice, because they are beautifully neat, but experienced fingers at once detect hard lumps and insufficient padding, which leaves a sharp edge uncomfortably prominent, although the calico or flannel may appear even from being securely sewn round.

There is no doubt that the perfect splint can only be achieved by patience and practice, but once it is accomplished, no inferior work will be put up with.

The well-made, firm, and even pad must be fastened very securely and well over-lapping the ends and edges of the iron or wooden splint. By remembering the purpose for which it is to be applied, its position with regard to the patient's limbs will be easily grasped, and a liberal margin of pad allowed, avoiding clumsy workmanship, of course.

When any material, which has served for padding, has to be used again, it must be thoroughly carbolised, and then dried slowly in front of the fire, and the greatest care taken that every scrap of it is sweet and clean, and picked over until it is, as nearly as possible, equal to new stuff.

The splint itself, whether of wood or iron, must invariably be scrubbed thoroughly and carbolised, even if it does not look particularly dirty.

If it is necessary to make the same linen, calico, or flannel covering do service a second time, it must not only be washed as carefully as the splint itself, and carbolic used, but it must be nicely ironed as well as dried.

Plenty of "heel" and other pads of various shapes and sizes should always be in readiness when a splint is ordered.

A well made and finished splint is the first necessity, but that, after all, is easier of attainment than the task which follows—of keeping it exactly in the position in which the surgeon has carefully adjusted it; and what nurse cannot recall dozens of incidents illustrating this puzzling point?

The lively little lad whose fractured thigh was caused by his falling from a wall, is sobered by the accident for a day or two only, and then he regains his normal spirits. And as he grows indifferent to the inconvenience of a broken leg, nurse's anxieties increase. She soon notices that when he thinks himself unobserved he wriggles and twists about a little, and perhaps next time she investigates his splint closely, she finds the bandage looks rather loose, for the boy's nimble fingers have been "easing" it a little, and now both sound and damaged leg prove equal to moving decidedly more than the surgeon suspects. Probably that bright-faced and innocent-looking boy possesses an instinct which advises him to present himself to the doctor in a reasonably good position, although he may occupy many of his waking hours in "wriggling and writhing"—of course, some of the movements may be harmless, but much of it can be stopped by a little judicious management, for there is frequently great irritation caused to a child's sensitive skin by the new flannel bandages or the strapping, and the confined position is very irksome.

If a child is found asleep with a little hand screwed in between his flesh and the bandage, it is well, after removing the intruding fingers, to see if there be any sign of heat or any rash, and a little starch powder or else dabbing with cooling spirit and water will often quiet the small mortal. Stray crumbs are a fruitful source of annoyance, and it is wonderfully difficult to banish all these, for they really seem to take a pleasure in hiding!

An occasional toy or portion of one is frequently bestowed about his person by the boy who objects to losing all his treasures for the night. How he contrives it no one knows, but he does manage to bury it in some obscure fold, from which it escapes later on, to become a source of discomfort to the sleeper and possibly of some danger to his skin.

### Presentations.

Miss Scholfield, who has been for 12 years the beloved and successful Matron of the Torbay Hospital, has, on her resignation been presented, with a pair of silver candlesticks, and a purse containing £180. Her departure is deeply regretted by all at Torbay.

The nurses of the East London District Nursing Society have presented their Matron, Miss Louise Taylor, with their photographs, handsomely framed together in one large frame. Last year they presented Miss Taylor with an afternoon tea-service and a toilette set. This shows the generosity of the nurses and the affection with which they regard one who has been over them so many years, and been a good friend to all.

Dr. Bond, for three years resident Medical Officer of the City Hospital, Grafton Street, Liverpool, was presented by the Matron and nurses with a silver inkstand, by the servants of the same institution, with a pair of candlesticks to match, as a token of their esteem and regard and expression of their regret at his departure, and wishing him every success in his new appointment as Medical Superintendent of Bradford Fever Hospital.

### Appointments.

Beccles Hospital.—Mrs. Oscar Granville Grayson, who trained at the Royal Infirmary, Edinburgh, has been appointed Matron to the hospital at Beccles, Suffolk, in place of Miss Macdonnell, who has left to undertake missionary work abroad.

Cambridge Infectious Hospital.—Miss Alice Taylor has been appointed Matron of this hospital for 18 beds and six cots. Besides general training, Miss Taylor has worked at the Leicester and Sheffield Borough Hospitals, the Colchester Infectious Hospital, and the Colchester Union Infirmary and the St. James's Union Infirmary. She has therefore special experience of fevers, and also of working under the poor-law; she ought to be the right woman in the right place.

Woodland's Home.—Miss R. S. Allen has been appointed Matron to the Woodlands Convalescent Home, Rawdon, Bradford. Miss Allen was trained at Birmingham, and has lately held the post of Matron at St. William's Hospital, Rochester.

### Nursing Uniforms.

### II.—GUY'S HOSPITAL.

GREAT is the fame of Guy's, both as a medical and a nursing school, and there are few of our readers, we are sure, who are not acquainted with its pretty uniforms.

First of all, the Sister, represented above in the middle figure, wears a gown of orthodox navy blue, white cap and apron, and outside collars and cuffs. Her cap is adorned with no adventitious strings or tails; it attains to dignity by means of severe simplicity, which must be a great comfort to its wearers.

Considering how tiring a nurse's life is, it seems almost cruel to put them in caps which prevent their ever leaning back with ease, or in caps, which, tying under the chin, cause unnecessary warmth in summer, and an ungraceful movement of the head at all times. The Sisters of the Middlesex Hospital have the best of it in this respect; their caps have loose strings, which are crossed well down on the breast, not tied up tight under the chin.

The lady pupils at Guy's have a distinct uniform of their own, and have distinct duties of their own. They belong to the race who perform no menial duties, and this, we suppose, is the reason they are allowed to wear black dresses with long insanitary trains. Guy's is built partially in blocks, and it looks very unprofessional to see a nurse sweep across a courtyard or gravel path and into a ward, taking the dust with her. But we confess that the uniform is graceful and pretty, the neat little three-cornered caps being worthy of all admiration; they are no trouble to get up, and they are easily put on, and very becoming. The right-hand figure represents the lady pupil.

Nurses at Guy's wear a lilac striped cotton gown, white cap, and apron. The cap ties under the chin. We want more Matrons like Miss Gordon, of Charing Cross, who has put not only all her probationers and nurses, but her Sisters into cotton frocks, which can be washed weekly. If there is any truth in the germ theory at all, then absolute cleanliness ought to be the first consideration in every nurse's uniform, and washing frocks ought to be rigorously insisted on.

### Death in our Ranks.

THE death is announced of Christina Watson, Queen's nurse at Bermondsey, from influenza. Miss Watson had acted as superintendent at Dublin and Bermondsey, and shown powers of management the cause can ill afford to lose.

### The Royal National Pension Fund for Nurses.

THE fifth general meeting of members of this Fund was held on March 3, when the annual report was submitted.

PENSION BRANCH.—During the twelve months there have been received 681 proposals (including 35 brought forward from last account) for granting pensions for £12,079 7s. 7d., of which 57 proposals, for assuring the sum of £1,046 16s. 11d., are still under consideration, or were not proceeded with; 624 policies were granted, for assuring £14 of immediate annuities, and £11,018 10s 8d. of deferred annuities, producing £3,952 6s. 6d. in single payments, and £6,768 8s. in annual premiums.

SICKNESS BRANCH.—In this department there have been received 169 proposals (including 9 brought forward from last account) for weekly sick-pay assurance of £114 12s. 6d., of which 41 proposals, for assuring the weekly sick-pay of £27, are still under consideration, or were not proceeded with; 128 policies were granted, securing £87 12s. 6d. weekly sick-pay, producing £147 12s. in annual premiums.

INVESTMENTS.—The investments on December 31, 1891, amounted to £106,517 1s. 5d. as follows: In British Government securities, £20,121 17s. 6d.; in foreign railway and other debentures and debenture stocks, £47,838 1s. 6d.; ditto shares (preference and ordinary), £5,806 16s. 10d.; in foreign Government securities, £18,843 12s. 1d.; in municipal corporation bonds and stocks, £3,767 2s. 6d.; investments of the Junius S. Morgan Benevolent Fund—in railway and other debentures, £9,034 10s. 6d.; in foreign Government securities, £1,105 0s. 6d.; total invested funds, £106,517 1s. 5d.

The foregoing figures are highly satisfactory, for they imply a steady and continuous growth in all branches of business. Six hundred and eighty-one proposals were received during the year, as against 524 in 1890, and 624 policies were issued, as against 436 in the previous year. The sickness branch is likewise progressing steadily, and has already been of material assistance to many nurses, as is amply shown by the many letters, full of expressions of gratitude, which have been received from those who have participated.

From time to time hostile critics have attacked the rates charged by the fund. But these rates have received the endorsement of the leading actuaries in this country, and in the United States of America; they are lower than the Post Office rates; they bear favourable comparison with those of other companies, having regard to the fact that the fund is a mutual one, and offers many special and substantial benefits to nurses which are not obtainable elsewhere. In these circumstances it is not surprising to find that nurses are learning more and more to choose the Royal National Pension Fund as the safest and most productive means of investment for their savings.

It is gratifying to know that the fund excites increasing interest amongst the philanthropic public. Large meetings, at which the objects of the fund were explained, have been held at Birmingham (under the presidency of the Mayor) and at St. Patrick's Hall, Dublin (under the presidency of Lord Justice Fitzgibbon). The Dublin meeting was called by Her Excellency the Countess of Zetland, and was attended by representatives of the chief hospitals and nursing institutions in Ireland.

It is a matter of considerable satisfaction to the Council that a movement has been set on foot in the United States to establish a National Pension Fund for workers amongst the sick on identical lines with those of the Royal National Pension Fund.

Above all H.R.H. the Princess of Wales continues to take the warmest interest in the progress and welfare of the fund. On July 25th she again received, at Marlborough House, about 600 nurses, representative of the second thousand who had joined the Fund. Permission was graciously given to many nurses who had been unable to be present on the former occasion to attend on this. Two days before the reception at Marlborough House, a conversazione was held at Merchant Taylors' Hall, which was generously lent by the master and wardens, when upwards of 1,500 nurses were received by the Countess Cadogan, one of the lady patronesses, as the representative of the President and Council of the fund.

The Junius S. Morgan Benevolent Fund, although it has only been in operation for a few months, has already been fruitful in benefits. It provides assistance for two classes of nurses: (1) For those who, being members of the Pension Fund, are unable from special temporary circumstances to keep up their payments or, it may be, even to maintain themselves; and (2) for nurses upwards of sixty years of age, not members of the Pension Fund, who are without sufficient means of livelihood. Altogether, sixteen nurses have benefited. Several who had joined the Pension Fund have been enabled to tide over difficulties, to take a much needed holiday in comfort, or to secure such other assistance as severe illness rendered it essential they should receive, with a view to their adequate treatment and ultimate recovery. When suffering from fatal illness the last few months of more than one nurse have been brightened by the timely and welcome grant which it has been possible for Lady Rothschild and her committee to make from the Benevolent Fund.

Mr. Walter H. Burns, in moving the adoption of the report, said: The report which the Council have placed before you does not call for any special comment from me. Its figures are very plain, and the steady progress of the Fund is clearly shown thereby. The past year has been uneventful for us in a certain sense, but it has been, on the other hand, one of uninterrupted prosperity so far as the objects of our Institution are concerned. There has been a considerable increase in the number of nurses who have taken our policies, and also in the Benevolent and other funds, together with a steady augmentation of our investments. I am happy to say that notwithstanding the events of the past year, which have been depressing to many institutions from the financial point of view, we have not a single loss of interest to record, and so far as the Council can judge, all our funds are invested in a thoroughly safe and satisfactory manner. During the past year the Princess of Wales has again shown her interest in the nurses by consenting to receive at Marlborough House the representatives of the Second Thousand who have joined the Fund, and I am sure I meet your wishes in placing on record in the fullest manner possible our gratitude to Her Royal Highness for this renewed proof of her interest and friendship in our cause. It is therefore with an increased sorrow that the Council on your behalf had to take upon themselves the sad duty of sending an address of sympathy to the Prince and Princess of Wales upon the death of the Duke of Clarence and Avondale. It was couched in the following words:—

At a meeting of the Council of the Royal National Pension Fund for Nurses, the following resolution was passed unanimously:—

The Council of the Royal National Pension Fund for Nurses, on behalf of the members of the Fund and the pensioners thereof, as well as upon their own, desire to offer to their Royal Highnesses the Prince and Princess of Wales their profound and respectful sympathy with the great bereavement they have suffered through the death of their eldest son, H.R.H. the Duke of Clarence and Avondale. The untiring interest which their Royal Highnesses—the Prince as patron, and the Princess as President of the Pension Fund—have shown in the cause of the nurses, and the help and encouragement they have given them render the grief and sympathy of the latter as keen as that of any other class of the community, while the nurses' familiarity with sickness and its attendant trials makes them more able perhaps than any class to appreciate the sorrow the Royal Family have experienced. The nurses and their Council humbly pray that the expression of their sympathy may not be unacceptable to their Royal Highnesses, and prove, if only in the slightest degree, a comfort to them in this hour of their tribulation.

On behalf of the Council of the Royal National Pension Fund for Nurses.

And I beg to add that we immediately received a gracious answer, reading as follows:—

Sandringham, Norfolk.

General Sir Dighton Probyn is desired to convey to the members of the Council of the Royal National Pension Fund for Nurses the warm thanks of the Prince and Princess of Wales for the sympathy they have expressed on the occasion of their Royal Highnesses' bereavement.

9th February, 1892.

The Royal Family has not been the only one that has suffered from the epidemic of influenza prevailing this winter, and if anything could add to the interest and appreciation of the work and devotion of nurses, it must be such special occasions for their aid as we have all recently passed through. Scarcely a family in the land but what has had some relative or friend stricken down and dependent upon the aid of these devoted women, for whom, I am glad to say, there is a steady increase of regard in the minds of all serious and well-thinking people in this country. Not only have they won this additional esteem from the patronage of the Royal Family and those placed in high social stations, but they have deserved it by the steady amelioration of their own class in knowledge, devotion, and attention to the sick, and untiring efforts to improve themselves. It is for this reason that I am again compelled to reiterate what has been said on every successive occasion of the meeting of the Council with its members, and that is that we cannot too strongly recommend hospitals to bestir themselves and to affiliate with our Fund, and thus aid in providing pensions for the nurses, many of whom are contributing more revenue to the hospitals than they receive from them. There is no doubt that in the minds of many there is, whether justly or unjustly, a feeling that the hospital authorities do not sufficiently provide for the future of their nurses, and while I should be extremely sorry to judge hastily, or to fail in recognising the good intentions of most of the hospital authorities, I cannot but feel that there is some ground for complaint by the nurses, especially in London. Many of the hospitals depend upon the goodwill and gifts of the public, and if they alienate the latter, they dry up the sources from which their own revenue comes. The Council of the Pension Fund has always been loth to divert from the hospitals directly to the nurses any of the gifts of the benevolent, but unless they can see on the part of the London officials more interest in securing pensions for their old servants, they may be compelled to make a more direct appeal to the public, which might possibly result in the hospitals getting less, and the nurses more of the public money. The quantity of funds to be distributed in charity and good works throughout the year is unfortunately not unlimited, and during the last year or two has not been an increasing one. There are many ways of affiliating with the Pension Fund devised to suit varied conditions and requirements. One of the simplest is that which has been adopted by the Leeds Trained Nurses' Institution, viz.: their Committee pay an annual sum for the benefit of nurses who have been ten years in their service, and who are policy-holders of our Fund. When their pensions become due, £10 a-year is added by the Leeds Nursing Institution to each pension out of the Trust Fund thus formed. In the report you will observe that there is an allusion to criticisms which have attacked the rates charged by the Fund, and to the statements that they are unreasonably high. As I represent, to a considerable extent, the givers of the Bonus and Benevolent Funds, about 2-5ths of which have been contributed by members of my family, I have looked carefully, on their behalf as well as my own, into these criticisms, and it seems to me that they are based upon an entire misconception of the subject. They leave out of consideration the fact that the Bonus Fund and all profits made are returned to the nurses, in addition to what they pay for themselves; and, besides, that most of the policies are upon a returnable basis—that is to say, the nurses or their families, at death or before, can withdraw all the funds which the nurses paid in, with interest at 2½ per cent. The pension rates are calculated upon the basis of 3 per cent. per annum on the tables in use at the Post Office Savings Bank, with a loading of 5 per cent. on the rate to provide for the necessary clerical expenses of the business. All the members of the Committee give their work for nothing, and the disbursements are the mere actual outlays without which the business could practically not be conducted. Our investments yield us an average of about 4¼ per cent., the difference of 1 per cent. per annum appears, therefore, as a surplus earning; but as it will be returned to the nurses, together with all the interest earned on the Donation Fund, it is a mutual system, and therefore it is quite impossible for any company to compete with us which is conducted on the basis of making a profit. I feel entirely satisfied with the rates which have been established by our Actuary (Mr. King); and on behalf of the donors to the funds, whom I represent, I can only say that the investigations to which I have been challenged by the critics have only resulted in increased confidence in the soundness and economy with which the fund is managed in the interests of the class which it is established to benefit. If any company charges apparently lower rates it can only be an illusion, or else done at a loss which cannot go on, for we are organised on the basis of making no profit and tendering the assured a substantial present besides. Can anyone reasonably claim that such a rate is too high? I think there is no impropriety in assuming that, with the advice of the high financial authorities who are members of the Council, our facilities for making safe and lucrative investments are at least as good as those of any association represented by our critics, and I can say of my personal knowledge that no effort is spared to take the best possible care of all the funds entrusted to the custody of the Council. I desire, before closing these remarks, to offer the thanks of the Council to Lady Rothschild and the other ladies who have taken charge of dispensing the benefits of the J. S. Morgan Benevolent Fund. It has already been of much service in relieving distress among nurses, especially members of the Fund, and will, I am sure, extend its usefulness more and more every year. Each case presented has been investigated with care, and Mrs. Roberts, the Acting Secretary of the Fund, has been specially zealous and devoted in her efforts to dispense its charities with liberality and judgment.

Mr. Burdett: I second this resolution. In doing so, I should like to point out two things. First of all, this institution is now in a position to give away £2,000 a year in benevolence to trained nurses, a fact which in itself places it apart from all other institutions, because there is no other institution existing in this country or in any other country which has power to give away an equal sum for a like purpose. Secondly, I want to point out that this is the only institution which enables nurses to insure for sick pay up to the age of 60 years, and which provides for them afterwards by pension. These are two very important points, which should certainly go forth to the public; and I am confident if they do go forth, they will show some of the special features of this Fund, and the value of it, in a more concise way than could be effected by any other two separate statements. I am sure, sir, we are indebted to you for your speech, and for your having commented at length on some points which have been raised against us. The fact that you should have taken such trouble, and gone over the criticisms as you did, should win for the Fund even greater confidence than it now possesses. I believe it has the confidence of all philanthropic institutions in this country, as well as of the nurses. It is a great fact that you should bring your large experience to bear on this matter, and, after going into all the details connected with the Fund, that you find everything satisfactory, and the criticism which I have mentioned absolutely without any solid ground. I have much pleasure in seconding the adoption of the report.

The meeting then unanimously agreed to the adoption of the report and the statement of accounts.

### The Council.

The Chairman said: The next matter is the election of five members of the Council in place of the four retiring. As I am one of these unfortunate victims, I think Mr. Burdett will have to take the chair.

Mr. Burdett: I do not think I will. I will leave you in the chair. I have much pleasure in moving that Mr. W. H. Burns, Dr. W. H. Broadbent, Mr. Thomas Bryant, Mr. Edward Rawlings, and Mr. Alfred Charles de Rothschild be re-elected members of the Council of this Fund. I am sure we are all very much indebted to Mr. Burns and all these gentlemen for the way in which they have helped us in this work. It is a great fact, and, I think, a very gratifying fact—and one that says a good deal for the City of London—that this National Pension Fund, which now holds in trust £50,000 for British nurses, is the outcome of the City of London entirely — £50,000 has been given out and out, free of all cost as to collecting, as an expression of gratitude from the City of London to the nurses of the British Empire. When we add that men whose names are recognised in the City as a tower of strength are giving their services and devoting their time to manage an institution of this kind—the magnitude of which is impressing itself more and more on the members of the Council, on whom the burden of the work chiefly lies—the fact is one which we ought to be thankful for, and of which we may be proud. I am myself not actively interested in the City, and, in fact, a neutral person, though connected with this Fund. Independently both of the value of your services, and the importance of the work, I think in contributing to the well-being of the nurses, to the well-being of our charities, you are contributing to the well-being of this country. Knowing how much time you give to this work, and how interested you are in it, and always prepared, even in the stress of business, to go into details, and to do anything that makes the Fund more efficient, I consider these circumstances should win gratitude and public recognition, as I believe they do outside this office altogether. I am not speaking these words as mere words of compliment, but very sincerely and with a grateful heart. I know such acts as yours are the very way to attract confidence and to gain public attention to the devoted services of the nurses and the needs which exist for a provision for their comfort when they are past work. And now we have got that provision, and it has become a practical fact, mainly through the efforts and labours of men like yourselves, we ought to recognise it on this occasion and to gratefully thank you for it. I have great pleasure in moving the re-election of the gentlemen whose names I have read.

Dr. Bristowe: I second the re-election of the retiring members of the Council.

The Chairman: You have heard the motion; Mr. Burdett must put it. Before he does so, I want to thank him personally for what he has said about myself. All I can say is that in what I have done I have only, in a feeble manner, tried to perpetuate what my lamented father-in-law started before me. I feel the deepest interest in the welfare of the nurses, and if I can aid them in any way, it will be a labour of love on my part to continue to do so.

Mr. Burdett: You have heard the resolution, and will signify your opinion in the usual way.

The resolution was then unanimously adopted.

### My Kitten.

I am a masseuse, and about half-a-dozen years ago I set up housekeeping in a third-floor front, and waited for the cases that were very slow in coming. However, owing to my strict attention to business and the punctuality and despatch that I showed in following the doctor's orders, things at last began to mend, and my furniture increased. In fact, after two struggling years, my increasing prosperity justified me in taking an extra room, and becoming the proud and happy possessor of a piano and a Chippendale bureau. *Seligkeit!*

Then it began to dawn upon me that such riches should be shared by someone. I wanted a companion. A being with a pleasant face to welcome me when I returned weary and footsore to my lofty dwelling. At first I thought of a husband. Then I thought of a cat—a nice homely, purring, affectionate pussy, and I at once began looking around to find that cat.

Some miles away from town, near the celebrated Hadley Woods, lived one of my patients who possessed a handsome Persian cat; and one day, after I had confided to her my desire to possess a real live pet, she kindly promised me one of Furry's kittens.

So in due time I got the loveliest little animal possible, with fur of a light chestnut—a rare colour for a Persian tom—and great sleepy eyes of a golden orange. Such a dear little soft face it had. I brought it up to town under my nurse's cloak, and at King's Cross Station I got quite frightened that someone would try to steal it, so covetous were the looks cast on it whenever it put its little head out to get a peep at the strange new world.

So Hadley Woods was brought to his home and was greatly astonished at going up so many stairs. All the other lodgers came up in turn to look at him, and told me awful tales about cats being stolen. Hadley Woods heard them with a contemplative expression on his wee face, but being of an affectationate disposition he took them all into his heart, and from that time forward was considered as the friend and protégée of everybody in the house.

He was almost too gentle, was that kitten; in fact, we began to think he wouldn't live long. The old lady in the kitchen shook her head over him, and I know, poor old soul, spent some of her rare pennies on buying cats'-meat for him, because it was strengthening. Then the second-floor lodgers commenced to be very fond of fish, and Hadley Woods loved fish better than anything in this world. I would see his little tail disappearing into the second floor front, the door being purposely left ajar, and one day he saved a haddock's fin for me, bringing it upstairs with pride, and depositing it at my feet.

But Hadley's life was not all play. I was determined that he should early understand that life was a serious thing, and that even a kitten could do something to make the world better and happier. I used to carry him with me on my rounds, and when we came to a patient who was lonely or unhappy, Hadley would remain to spend the day and amuse the invalid. He was such a happy, intelligent little fellow, they said, and always behaved so nicely. When anybody had a pain he was so sorry, and would put his wee face against the sufferer's and purr with all his might and main to make it better. I was not able to leave him with hysterical patients because he was too sympathetic, and he lacked the firmness of character required in dealing with these cases.

My doctors got to know Hads, and liked him. One of them had a little invalid daughter, who fretted so after my kitten that her father, to whom I owed many kindnesses, begged me to let her have it. So, sorely against my will, I carried him to the doctor's house, and left him frisking about with his squirrel-like tail, to the huge delight of the sick child. But it was very dull that evening in my little sitting-room. No kitten to sit on the hassock in front of the fire, or to jump on my shoulder and put its little damp nose in my ear with a sudden but affectionate purr. Reading was impossible, though the last number of The Hospital had just arrived; and appetite deserted me, though I opened a long-cherished pot of home-made jam for my tea. The very kettle knew something was wrong, and spit wrathfully all over the fender in an aggravating and unusual fashion.

I went to bed that night feeling that life was not worth living, and found that the mattrass was lumpy and the pillow hard—facts I had never discovered before. I fell asleep at last, only to awake in a few hours with a kitten's me-ow in my ear. I listened but could hear nothing, and went to sleep once more, but was awakened again with that faint me-ow. I jumped out of bed and opened the window; it was pouring with rain and very dark, but nothing could be heard excepting the rain-drops. Finally I put on some clothes and my shoes, pulled a shawl over my head, and went down to the door, but nothing was to be seen.

I don't know what possessed me to go along the street, half-dressed as I was; but I did so. Another street crosses ours a few paces up, and at the junction I stopped and listened. All at once I heard a piteous me-ow at my feet, and a little damp ball pressed against my skirt. Poor little Hads, he had waited for me to fetch him home, and, finding I did not come, had crept out when the house was quiet to find his way back alone. He had managed to get as far as the cross streets, and then, bewildered and weary, had sat down and me-owed for me. How it was that I heard at that distance I did not know till I read Mr. Stead's Christmas Annual for last year. Now I feel sure that Hadley has an astral voice.

So I carried him home, a wet and draggled, but happy little fellow. I was very much afraid he had caught cold, and thought I had better put his feet in hot water and mustard, but dismissed the idea as foolish. He was well rubbed with a towel instead, and I made some milk hot over the lamp and gave him to drink; then he tucked himself under the eider-down and went to sleep.

He did not catch cold and die, but as I write a great cat, with long red fur, sits and gazes at me with sleepy, adoring eyes. Hadley believes that there is nobody half as clever or as good or as beautiful as I am, and it is pleasant to be loved and admired, be it only by a cat.

### Notes and Queries.

### Queries.

*Paraffin Stain.*—Can anyone tell me what will remove paraffin from a carpet?—*C. E. J.*

*Book about Drugs.*—Will someone please tell me the name, publisher, and price of a book about common drugs. What they are derived from, &c.?—Lizzie.

### Answers.

*Dimple.*—Your indoor uniform will be quite suitable if your cap is a flat one on which the Bishop can conveniently lay his hands. If it is not, wear a flat one for the occasion.

*Vincent.*—(1) You must go to Doctors' Commons, where, on payment of a shilling, you will be shown a copy of the will. (2) Both the same.

*Nurse Brown.*—Certainly not; how can you listen for a moment to such rubbishy gossip. See The Hospital for July 27th, 1889, for a life of Miss Nightingale.

*Lily.*—There are so many good cheap books on nursing, you had better ask the Matron of the hospital to which you are going to recommend one to you. Failing this, get Lewis's "Theory of Nursing," price 3s. 6d. from this office.

*Sister Dora.*—We shall be pleased to see you during your holiday. Go to Miss Culverhouse, 18, Royal Avenue, Chelsea; terms about 18s. a week, house very comfortable, close to "Venice in London."

*E. B.*—The cheapest nursing home we know is Mrs. Power, 29, Upper Montagu Street, W. Or you can get a bed in a ward at Guy's for a guinea a week.

*Nurse Grey.*—We were very pleased to get your letter, and we congratulate you.

## Figures and Tables

**Figure f1:**